# Effect of Fortified Feed with Phyto-Extract on the First Physical Barrier (Mucus) of *Labeo rohita*

**DOI:** 10.3390/ani11051308

**Published:** 2021-05-01

**Authors:** Francesco Fazio, Saira Naz, Syed Sikandar Habib, Mehmood Ahmed Husnain Hashmi, Muhsin Ali, Concetta Saoca, Mujeeb Ullah

**Affiliations:** 1 Department of Veterinary Sciences, Polo Universitario Annunziata, University of Messina, 98168 Messina, Italy; csaoca@unime.it; 2 Institute of Molecular biology and Biotechnology, University of Lahore Sargodha Campus, Sargodha 40100, Punjab, Pakistan; saira.zoologist1991@gmail.com; 3 Department of Zoology, University of Sargodha, Sargodha 40100, Punjab, Pakistan; sikandarzoo00@yahoo.com; 4 Department of Zoology, Wildlife and Fisheries, University of Agriculture, Faisalabad 38040, Punjab, Pakistan; mehmoodhashmi755@gmail.com; 5 Department of Zoology, Hazara University, Mansehra 21120, KP, Pakistan; muhsinalizoologist@gmail.com; 6 Department of Zoology, Islamia College University, Peshawar 25120, KP, Pakistan; mujibkhanicp@gmail.com

**Keywords:** *Labeo rohita*, *Zingiber officinalis*, *Withania coagulans*, fish mucus, fortified fee

## Abstract

**Simple Summary:**

Pathogens of fish are a serious issue faced by farmers and a great threat to the country’s economy. Overuse of antibiotics leads to antibiotic-resistant strains of the bacterial pathogens; residues of the antibiotics also accumulate in the tissue of the fish and are responsible for environmental problems. Therefore, the use of antibiotic alternatives should be explored as a new approach to immunotherapy to prevent or to cure preexisting infections. Previous research has concluded that the use of herbal extracts enhances the immunity of fish against several fish pathogens such as bacteria and other protozoon parasites. This study aimed to evaluate the effect of two different fortified feeds with different concentrations of *Withania coagulans* and *Zingiber officinale* on the fish mucus of *Labeo rohita.* The mucus was tested against five pathogenic bacteria in-vitro while fish was tested against the ectoparasite Lernaea (in-vivo). Our result showed that *Z*. *officinale* proves an efficient immune stimulator for the *L. rohita* against the tested organism (both in vivo and in vitro). Hence, it can be used as an effective solution against the emerging diseases of cultured fish.

**Abstract:**

The aim of the current study was to assess the effect of two different fortified feeds with different concentrations of two important medicinal plants (*Withania coagulans* and *Zingiber officinale*) on the mucosal immunity of *Labeo rohita.* After a dietary intervention, mucus was tested against five pathogenic bacteria (in-vitro), while experimental fish were tested against the ectoparasite (Lernaea) (in-vivo). Our results revealed that all fish groups fed with different concentrations (1, 1.5, and 2%) of *Z. officinale* had low molecular weight proteins and did not develop any significant signs of parasitic infection, with low mortality rate; whereas the groups that were fed with *W. coagulans* (particularly with 1% and 2%), including a control group, developed rapid signs of infection with high mortality rate. The highest hemagglutination titer value was recorded for the fish fed with 1% and 1.5% of *Z. officinale*. The lowest value was found for the fish fed with 2% of *W. coagulans*. The mucus of all fish of fortified groups was active and inhibited the growth of tested bacterial pathogens as compared to the control group. Further, *Z. officinale* groups showed greater efficacy against bacteria as compared to the *W. coagulans* groups. In conclusion, *Z. officinale* can be considered as a potential and functional ingredient in aquaculture feed. Furthermore, future studies should be conducted to investigate more details on the subject.

## 1. Introduction

The fisheries sector plays an important role in livelihood, food security, and economy of a country, in particular in developing countries [1]. The increased demand for fish protein and advanced technologies represents the main reason for the growth of the relative industries. Due to the expansion of this sector, aquaculture practices have become more concentrative for high yield production [2]. Aquaculture farmers face a great problem such as diseases due to several pathogens like virus, bacteria, and other parasites; in fact, almost 50% of production losses is due to these pathogens [3]. Finfish are the host of a great variety of ecto- and endoparasites [4]. Although these parasites generally do not affect the fitness of healthy fishes, they can become a dangerous problem under stressful conditions, which mostly occur in captivity; on the other hand, several bacterial pathogens such as *Vibrio anguillarum, Vibrio vulnificus, Aliivibrio salmonicida,* etc. are responsible for deadly infections [5]. Currently, several treatment strategies have been implemented, such as the use of antibiotics and other chemicals like copper, acriflavine, and formalin, etc. There are several risk factors associated with these chemicals such as the potential harmful effects to consumer health and direct toxicity to fish and the environment; therefore, their use is prohibited in aquaculture [6]. For this reason, in recent years, the search for disease control methods that are not harmful to the environment has increased. Infections and stress cause the suppression of the immune system of the host [7], which results in a weakening of the defense system and an increase in susceptibility to many diseases. An alternative approach is to stimulate the overall immunity and health of fish to prevent disease [8]. Several medicinal plants, such as olive leaves extract, have been used as additives to enhance the immunity and health of fish [9]. In fish, the skin and its associated mucus secretions act as a first barrier to pathogens and have a major role in fish immunity [10]. Several previous studies were conducted on the use of feed additives to improve fish mucus properties; for example, the use of Persian hogweed (*Heracleum persicum*) as feed additives in common carp feed, significantly increases several immunological factors like proteases, lysozymes, and alternative activities of complement [11,12]. It was also shownthat the peppermint extract used in the diet of Caspian brown trout (*Salmo trutta caspius*) and Caspian white fish (*Rutilus frisii kutum*) increased resistance against pathogens [13].

Medicinal plants used in the current study have several therapeutic properties such as extraction of *Withania coagulans* is well known for its many pharmaceutical properties. This fruit belongs to the family Solanaceae that is famous for its many therapeutic activities. Its extraction has shown many miracles from accent time like the healing of the wound, preventing high blood sugar (antihyperglycemic), preventing cancer, and enhancing the immunity of the species against different infections [14]. It also reduces inflammation and oxidative stress [15]. Moreover, ginger (*Zingiber officinale*) belongs to the family Zingiberaceae. They are commonly available in East and Southern Asia. This family comprises 49 genera with 1300 species. *Z. officinale* contains several phenolic compounds like shogaols, gingerol, gingerdione, shogaols, and gingerdiol. Besides these phenolic compounds, flavonoids, paradols, sesquiterpenes, minerals and vitamins are also present. Ginger has been in use for a long time for the treatment of cold symptoms, stomach pain, vomiting, fever, dementia, cramps, sore throats, etc. It also possesses several other therapeutic properties like anti-tumor, anti-inflammatory, anti-apopotic, anti-platelet, anti-pyretic, anti-diabetic, anti-hyperglycemic, and anti-oxidant [16]. Despite several therapeutic properties, the use of *W. coagulans* and *Z. officinale* in aquaculture is very limited, therefore, the current study aimed to evaluate the potential benefit of these two magical herbs in aquaculture as an immune stimulant.

## 2. Materials and Methods

### 2.1. Study Site and Duration

The study was conducted in the fisheries department Chashma, Mianwali for 30 days. Rohu (*Labeo rohita*) was selected as the experimental animal. This study is registered under trial no FDM/LR:321. All experimental procedures were carried out under European legislation regarding the protection of animals used for scientific purposes (European Directive 2010/63).

### 2.2. Feed Preparation and Fortification

The *W. coagulans* specie was collected from Village Tarki Khel of district Karak, KP Pakistan, which grows as a wild plant. The specie was identified by the botanist. After drying, the fruit of *W. coagulans* was ground into powder form by using an electric grinder (SAMFORD Germany Automatique Series model: SG 8011) and stored in the clean sterile bottle away from the direct sunlight and moisture while the *Z. officinale* was purchased from the local market.

After peeling, ginger was cut into fine pieces and dried in sunlight for 6 days. After completely dried, ginger was powdered by using an electric grinder (SAMFORD Germany Automatique Series model: SG 8011). Then it was sieved and stored in the air-tight container away from the direct sunlight and moisture until further use. The commercial feed of known ingredients and nutritional value was fortified with different concentrations of the *Z. officinale* and *W. coagulans* that is 1%, 1.5%, and 2% concentration in diet and then spread for 24 h on different shaped trays and dried at room temperature. The proximate composition of the diet was analyzed using the protocol of AOAC [17]. After analysis, it was revealed that the diet contains an average dry matter of 89.58–90.5%, crude protein 35.00–35.98%, lipids 4.9–4.16%, total ash 7.72–7.89%, moisture 10.18–10.28%, and crude fibers 2.20–2.27%. The fortified feed was stored in a container with closed lids and placed in a dry and ventilated area at a temperature of 5–6 °C. The extract composition of both plants extracts was analyzed with the help of Liquid and Gas chromatography (HPLC and GC) by using UV detector and FID respectively. Table 1 summarizes all the extract compositions of both plants and Table 2 shows fortification detail.

### 2.3. Rearing of the Fish

Clinically healthy (no sign of infection) fish were caught with the help of a local fisherman at Chashma barrage. After weighing (433 ± 213 g), each fish was bathed with the KMnO_4_ solution (8.1 ppm) to prevent infection of pathogen or any other contamination. Before starting the experimental procedure, fishes were divided into four groups in the circular tanks made up of cement and acclimatized for 15 days to normalize the function of the fish body. During the period of acclimatization, the only basal diet was served. Grouping detail is given in Table 3.

During the whole course of study, water quality parameters were checked daily with the help of DO meter (Hanna Instrument HI9132), pH meter (Hanna Instrument HI221), and thermometer. Mean water temperature was recorded as 27 ± 0.6 (°C), DO as 5.7 ± 0.03 mg/L, and pH was 7.3.

### 2.4. Mucus Collection

On the termination of the experiment, fish were anesthetized with MS-222 and the selected fish was smoothly laid down on the surface of the table. With the help of a plastic spatula, the surface (dorso-ventral) of the fish was wiped gently. Strick precautions were carried out during mucus collection to prevent cross-contamination like scales, blood, and intestinal fluids. On average, 3–5 mL of mucus sample was collected from each fish. Collected mucus samples were centrifuged at 4 °C at 12,000 rpm for 10 min to selectively draw subcellular organelles into the pellet. The supernatant was then collected in the sterile vials and stored in the freezer at −40 °C until further experimental procedures.

### 2.5. Total Protein Content

Before further analysis, the samples were thawed at room temperature. Then for the determination of protein contents in the mucus, Bradford Micro Assay technique was followed, while Bovine Serum Albumin (BSA) was used as standard. In this process, both BSA and the sample of mucus were diluted in de-ionized water in microtiter plates with a flat bottom. After that, Bradford protein solution of 50 µL was poured into the wells while absorbance was recorded at 595 nm. A standard curve was drawn while protein concentration was determined by comparing their readings with the standard BSA solution curve as described by [18,19].

### 2.6. Molecular Weight of Proteins

For the determination of molecular weight, polyacrylamide gel electrophoresis (SDS-PAGE) was used as described by Laemmli [20], but with few adjustments. In this process, under 120 constant voltage, 15% separating and 4% staking buffer were used to operate the SDS-PAGE. For the non-reducing protein, the Fermentas PageRuler™ protein ladder was used as a standard marker. The gel used in the gel-electrophoresis was stained with Page Blue.

### 2.7. Challenge Study

After feeding the fish with Z. *officinale-* and *W. coagulans*-enriched diet for 30 days, then 20 fish were kept separately and challenged with the ectoparasite (Lernaea) and continued feeding for further 30 days. Clinical signs of infections and the presence of parasites were thoroughly checked from randomly selected three fish fortnightly [21]. Caught fishes were kept alive in the bowls made of plastic. If Lernaea was present, then it was removed with the help of fine forceps and preserved in the 5% formalin and then permanently mounted for identification using published keys [22]. Further, cumulative mortality and relative percent survival (RPS) were counted according to the equation of Amend [23].

$$\mathrm{Cumulative}\;\mathrm{Mortality}=\frac{total\;mortality\;in\;each\;treatment\;\;after\;challenge\;}{Total\;number\;of\;fish\;challenged\;for\;the\;same\;treatment}\times 100$$

### 2.8. Lectin Activity

This activity was determined by following the method of Ewart et al. [24]. In this procedure, a sample of 50 µL was diluted in phosphate buffer (PBS) with pH 6.2 and 0.05 M double-folded in the microtiter with a U-shaped bottom. As a control group, 50 µL rabbit RBC solution was thoroughly mixed with the dilution and incubated for 1 h at room temperature. After that, for the determination of hemagglutination (HA), a plate surface was used. HA titer can be defined as the reciprocal of the highest dilution exhibiting hemagglutination, was computed as one HA unit (mg^−1^ lectin) [25].

### 2.9. Alkaline Phosphate Test

The test mucus sample was incubated in 100 mM ammonium bicarbonate with 4 mM p-nitrophenyl phosphate and 1 mM MgCl_2_ (7.8 pH and 30 °C). Optical density was measured for 3 hours continuously using a microplate reader at 405 nm. The starting of the reaction was used to calculate the activity while one unit (U) of activity can be defined as a concentration of the enzyme required to release 1 µmol of p-nitrophenol product in 60 s [26].

### 2.10. Disc Diffusion Method (In-Vitro Antimicrobial Activity)

The extracted mucus was evaluated against five bacterial pathogens such as *Escherichia coli, Flavobacterium columnare, Edwardsiella piscicida, Proteus mirabilis,* and *Salmonella paratyphi*. Bacterial pathogens were obtained from the microbiology department of Kohat University of Science and Technology. In the disc diffusion method, 0.1 mL of the tested bacterial strains were spread on the plates of the agar surface and were grown in the Muller Hinton Broth medium at a temperature of 37 °C for 24 h. Suspension of the bacterial concentration was adjusted to 10^8^ CFU/mL (colony-forming unit/mL). Six-millimeter diameter paper discs were impregnated on the agar to load 10 microliters of mucus sample. The impregnated discs were placed on the medium that was properly at distance and then the plates were incubated for 1 hour at 5 °C; this incubation was done to diffuse the sample properly and then further incubate in the incubator for 24 h at 37 °C. After that, the zone of inhibition was measured. The experiment was performed in triplicate.

### 2.11. Statistical Analysis

For statistical analysis, SAS software version 9.1 was used. ANOVA was applied to the findings (significance level *p* < 0.05). Means were compared by using Duncan’s multiple test.

### 2.12. Ethical Approval

This study is registered under trial no FDM/LR:321. All experimental procedures were carried out under European legislation regarding the protection of animals used for scientific purposes (European Directive 2010/63).

## 3. Results

### 3.1. Protein Profiles

**Figure 1:**
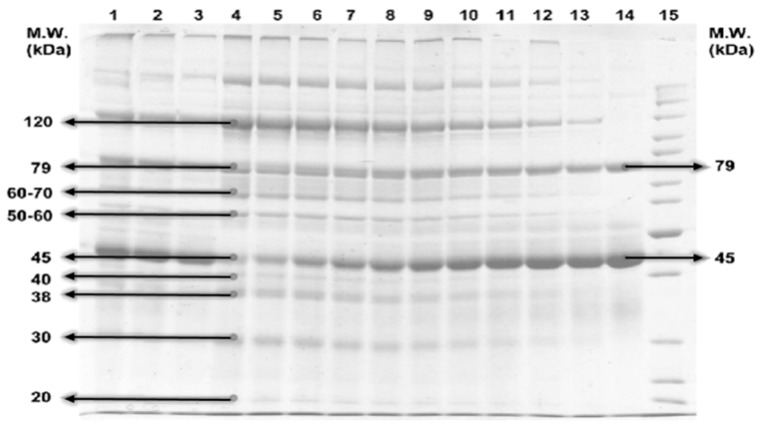
SDS page.

After the SDS page run, it was revealed that G2-WC 1% and G4-WC 2% have high molecular weight proteins (100 kDa and 120 kDa respectively) as compared to the other groups. G4-ZO 2%, G2-ZO 1%, and G3-ZO 1.5% have low molecular weight proteins (14.68 kDa, 20.32 kDa, and 14.00 kDa respectively). Details are given in Table 4; Figure 1 shows the SDS page.

### 3.2. Challenge Study (In-Vivo)

**Figure 2:**
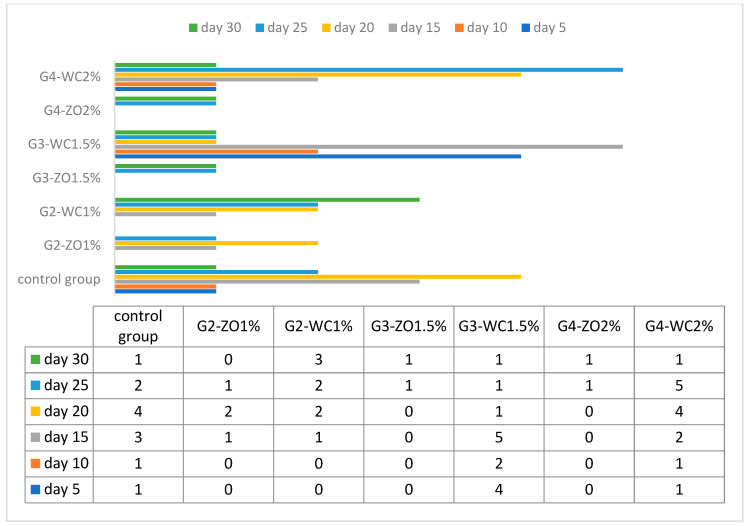
Mortality rate after challenge.

After introducing Lernaea parasite, it was found that fish of group G4-ZO 2%, G2-ZO 1%, and G3-ZO 1.5% did not develop signs of any infection and, therefore, they were resistant to the parasites. Instead, the groups CG, G2-WC 1%, and G4-WC 2% developed rapid signs of infection. Table 5 shows details of the experiment and Figure 2 illustrates the mortality rate.

The highest HA titer value was recorded for the group G2-ZO 1% (2^9^) and G3-ZO 1.5% (2^9^). The lowest value was found for G4-WC 2% (2^6^). On the other hand, the highest value of ALP was obtained in G2-WC 1% (161.3 ± 0.4^b^) and G4-WC 2% (152.5 ± 0.7^d^). Details can be seen in Table 6.

### 3.3. Disc Diffusion Method (In-Vitro)

When the extracted mucus was tested against the bacteria in laboratory conditions, it was showed that fish fed with 1% and 1.5% of *Z*. *officinale* were more active against the bacterial pathogens, in particular against *E. piscicida* and *S. paratyphi*. Further details of this experiment can be seen in Table 7.

## 4. Discussion

All living organisms have a strong system of defense as a technique of survival against pathogens that cause diseases. The complement system of the fish is composed of cell surface components and more than 32 serum proteins. This immune system is responsible for the recognition and elimination of the pathogen as the acquired and innate immune system [27]. Fish skin mucus is a very dynamic biological defense line system between the environment and fish. The mucus of the fish is composed of biochemically active secretions from the goblet of the epidermis and epithelial clavate cells [28]. These cells are responsible for the secretion of gelatinous macromolecules known as mucins and water, which eventually transfer into mucus [29]. This skin mucus has to face the challenges of potential deadly parasitic attacks, and it also prevents the various parasite colonizations.

The current study was focused on evaluating the effect of two different types of plant-based feed additives (*Z. officinale* and *W. coagulans)* on the mucus protein profiling of *Labeo rohita*. The results revealed that G2-WC 1% and G4-WC 2% have high molecular weight proteins while G4-ZO 2%, G2-ZO 1%, and G3-ZO 1.5% have low molecular weight proteins. In the other groups, protein weight falls in between these two extremes. If comparing the level of parasite infestation/mortality rate with the concentration/molecular weight of protein, it was found that the groups with high concentration/molecular weight (G2-WC 1% and G4-WC2%.) have high mortality/infestation rate. The high concentration/molecular weight seems, therefore, that it is inactive against the Lernaea. This is probably due to the fact the high-concentration proteins represent a convenient source of food for the parasitic population, which can consequently proliferate drastically. The groups that have low molecular weight and low concentration proteins also have a low mortality/infestation rate, such as the group G-3-ZO has distinctly low molecular weight protein (14.00 kDa), which is quite close to lysozymes (14.00 kDa–15.00 kDa), and has a large number of antimicrobial properties [30]. These lysozymes contribute to the innate immune system of the organism and are more active against gram-positive bacteria thanks to the presence of peptidoglycan in their cell wall [31], which is a target region that hydrolyzes the glycosidic bond. In many fishes, the role of the lysozymes as a catalytic agent is very prominent. Besides this, G3-ZO 1.5% and G4-ZO 2% have a molecular weight of 31 kDa, which indicates the presence of carbonic anhydrase, which plays an essential role in the innate immune system. In a previous study on *Labeo rohita,* protein from the mucus was isolated and the results showed a value of 0.55 mgm/L, which is lower than that of our study. This may be due to different environmental conditions and the food consumed [32]. The difference in the protein content concentration recorded in the different studies [33,34,35] may be due to mucus collection techniques, protocols followed, age difference, sexual maturity, environmental stress, seasonal variation, and difference in the living environments (water quality parameters).

Another defense mechanism present on the surface of the fish body is the availability of the lecithin protein (HA titer) [36]. High values of HA titer indicate that fish is under stress due to exposure to diseases or water from the contaminated area [37]. In our study, G2-ZO 1% and G3-ZO 1.5% have higher values for the HA titer, which show maximum life span as compared to others. Several other studies were conducted to elevate HA value in different fishes like. Ng et al. [38] showed a value of 2^7^ for grass carp; Sahoo et al. [39] determined the values for grass carp (2^6^) and *Labeo rohita* (2^9^). Values for the *Labeo rohita* are similar to our findings.

Another stress indicator in fishes is the alkaline phosphate activity (ALP), which plays an important role in the immune system. High values of ALP were observed in injured or diseased fish [40]. Omolbanin et al. [41] in their study evaluated the ALP value of common carp and found that this value was higher in older, infected or injured fish. In our study, G1-WC 1% and G4-WC 2% showed a higher value of ALP, in fact they suffered from the infection and had a high mortality rate respect the other groups. Further increased value of the ALP is an indicator of stress.

As well demonstrated, the mucus of the fish skin is a perfect barrier against surrounding pathogens [42]. Moreover, in addition to tapping and eliminating deadly pathogens, it represents a reserve of antimicrobial peptides, which act in many different ways [43]. The evaluation of mucus extract against different bacterial pathogens carried out in our study, showed that all fish that were fed with *Z*. *officinale* inhibited most of the bacteria with a large zone of inhibition in respect to the groups fed with *W. coagulans*. Furthermore, group 2 (G2-ZO 1%) was more active against *Salmonella paratyphi,* while the same group in G2-WC 1% did not effectively inhibit the growth of the bacteria. However, overall, fortified feed showed greater efficacy against the same bacteria in respect to the control group. The minimum inhibition for *E. coli* was achieved by the control diet, with an efficient inhibition by the fortified diets, particularly by that with the *Z*. *officinale.* Previously, Bragadeeswaran and Thangaraj [44] reported that the crude extract of mucus from *Anguilla anguilla* shows strong antimicrobial activity against *P. aeruginosa, E. coli,* and *S. aureus,* while no activity was shown against *K. pneumonia.* Surprisingly, the current study shows a strong anti-microbial activity of all groups fed with *Z*. *officinale* in respect to those with *W. coagulans.* Though both of the medicinal plants exhibit strong antimicrobial activity [45,46], this point should be investigated at the molecular level in more detail.

## 5. Conclusions

In the current research, it was demonstrated that the extract of two medicinal plants is effective in improving the natural defense system (skin mucus) of *Labeo rohita*. This is a preliminary study on the effect of two important medicinal plants on mucosal immunity in aquaculture. Between these two plants, *Z*. *officinale* shows satisfactory results and can be considered as a potential and functional ingredient in aquaculture feed. Furthermore, future studies should be encouraged to investigate more detail.

## Figures and Tables

**Table 1:** Extracts from the experimental plants.

*Withania coagulans (WC)*	*Zingiber officinaleis (ZO)*
Main Compounds	Main Compounds
Glycosides	Paradol
Steroidal compounds	Shogoal
Saponins	Zingerone
Phenolic compounds	Zerumbone
Tannins	1-Dehydro-(10) gingerdione
Triterpenoids	Terpenoids
Flavanoids	Ginger Flavonoids
Alkaloids	

**Table 2:** Fortification of the diet.

Basic Ingredients (g/100 g)	Diet			
	Basal	1%	1.5%	2%
Soybean meal	35.2	35.2	35.2	35.2
Wheat offal	7	7	7	7
Ca (PO4)2	0.2	0.2	0.2	0.2
Palm oil	0.4	0.4	0.4	0.4
Fish meal	21	21	21	21
Vitamin and mineral premixa	1	1	1	1
Common salt (NaCl)	0.2	0.2	0.2	0.2
Maize powder	35	34	33.5	32
*Z. officinalis* or *W. coagulans*	00	1%	1.5%	2%
Total	100	100	100	100

**Table 3:** Group categorization and feed details.

	G1 Group	G2 Group (1%)		G3 Group (1.5%)		G4 Group (2%)		Total
Sub Groups	G1-control	G2-WC1%	G2-ZO1%	G3-WC1.5%	G3-ZO1.5%	G4-WC2%	G4-ZO2%	
Fish quantity (*n* =)	30	30	30	30	30	30	30	210
Challenge Study	20	20	20	20	20	20	20	140
Weight (g)	433–436	430–432	439–435	432–437	432–439	432–439	431–438	435 Mean

**Table 4:** Protein profiling of the groups.

	G-1 (Control Group)	G-2		G-3		G-4	
		(G2-ZO1%)	(G2-WC1%)	(G3-ZO1.5%)	(G3-WC1.5%)	(G4-ZO2%)	(G4-WC2%)
Protein Profiling (kDa)							
1	89.12	40.32	90.25	14.00	90.3	14.68	21.54
2	85.13	20.32	100.00	75.64	89.6	20.19	18.00
3	75.12	25.25	81.2	13.68	87.65	45.56	120.36
4	58.87	30.32	98.79	20.65	45.8	54.98	13.54
5	100.63	36.3	95.6	21.63	99.8	78.65	18.98
6	101.25		98.65	31.08	84.21	31.00	45.69
7	120.23		90.3		75.69	14.28	75.65
8	81.23		75.5		78.54	13.56	78.32
9	33.12				90.58		
10	36.45				46.35		
11	12.36						
12	17.56						
13	50.13						

**Table 5:** Clinical observation of the infections.

Groups		Day 7	Day 14	Day 22	Day 30	Cumulative Mortality %
G-1	CG	-	+	++	+++	60
G-2	G2-WC 1%	-	-	+	++	20
	G2-ZO 1%	-	-	+	-	5
G-3	G3-WC 1.5%	+	-	+	++	15
	G3-ZO 1.5%	-	-	-	-	10
G-4	G4-WC 2%	+	+	++	+++	25
	G4-Z O2%	-	-	-	-	5

Note: (-) no sign, (+) low level, (++) moderate level and (+++) high level of infection or infestation.

**Table 6:** Values obtained for ALP, HA titer, and Protein concentration.

Groups		Concentration of Protein	HA Titer Value	ALP
G-1	CG	3.63 ± 0.48 ^a^	2^8^	86.5 ± 0.7 ^a^
G-2	WC 1%	3.00 ± 0.07 ^a^	2^8^	161.3 ± 0.4 ^b^
	ZO 1%	2.59 ± 0.08 ^b^	2^9^	53.3 ± 0.56 ^c^
G-3	WC 1.5%	2.02 ± 0.57 ^b^	2^7^	89.6 ± 0.2 ^d^
	ZO 1.5%	2.68 ± 0.48 ^b^	2^9^	18.29 ± 0.1 ^e^
G-4	WC 2%	2.29 ± 0.13 ^b^	2^6^	152.5 ± 0.7 ^f^
	ZO 2%	1.98 ± 0.02 ^c^	2^7^	148.25 ± 0.45 ^g^

Note: Numbers with the same superscripts in the same column are not significantly different (*p* > 0.05).

**Table 7:** Zone of inhibition against the tested bacterial pathogens.

Bacterial Strains	Zone of Inhibition (mm)						
	Control	G2		G3		G4	
		G2-ZO 1%	G2-WC 1%	G3-ZO 1.5%	G3-WC 1.5%	G4-ZO 2%	G4-WC 2%
*E. coli*	18.65 ± 0.24	30.58 ± 0.67	23.46 ± 0.32	30.65 ± 0.69	24.36 ± 0.45	30.48 ± 0.48	20.14 ± 0.21
*F. columnare*	19.64 ± 0.45	30.57 ± 0.98	24.65 ± 0.45	36.35 ± 0.89	26.75 ± 0.75	33.45 ± 0.79	28.36 ± 0.31
*E. piscicida*	20.78 ± 0.14	31.23 ± 0.89	20.54 ± 0.36	38.45 ± 0.24	26.34 ± 0.45	34.15 ± 0.69	29.31 ± 0.24
*P. mirabilis*	25.32 ± 0.19	28.54 ± 0.87	24.65 ± 0.78	30.32 ± 0.75	21.31 ± 0.48	32.24 ± 0.36	26.34 ± 0.24
*S. paratyphi*	20.14 ± 0.65	39.72 ± 0.47	25.40 ± 0.45	31.21 ± 0.69	25.45 ± 0.47	33.24 ± 0.48	28.35 ± 0.14

## Data Availability

The data presented in this study are available on request from the corresponding author.
